# Sudden Gross Visual Deterioration: Importance of Examining the Whole Eye

**DOI:** 10.7759/cureus.34374

**Published:** 2023-01-30

**Authors:** Naeem Iqbal, Samantha R De Silva, Susan M Downes

**Affiliations:** 1 Department of Ophthalmology, Queen Alexandra Hospital, Portsmouth, GBR; 2 Department of Ophthalmology, John Radcliffe Hospital, Oxford, GBR; 3 Department of Clinical Neuroscience, University of Oxford, Oxford, GBR

**Keywords:** medical education, acute visual loss, low vision, diabetic retinopathy, lens dislocation

## Abstract

A 75-year-old caucasian female presented with sudden severe visual deterioration in one eye reduced from 6/9 to counting fingers (CF), with second eye reduction in vision from 6/9 to CF three months later. Past medical history included a background of proliferative diabetic retinopathy, uncontrolled blood pressure, and a 44-year history of poorly controlled type 1 diabetes mellitus (T1DM). Previous ocular history included bilateral pan-retinal photocoagulation for proliferative diabetic retinopathy, followed by bilateral vitrectomies, with subsequent bilateral cataract surgery with intraocular lens implants. A diagnosis of anterior ischemic optic neuropathy (AION) was thought to be the most likely diagnosis due to sudden visual loss, pale discs, and previous long-term history of diabetes and blood pressure with variable control in the absence of a raised erythrocyte sedimentation rate (ESR). However, at the time of the second eye visual loss, the inferior peripheral retina examination revealed bilateral pseudophakic intraocular lens dislocations. With spectacle correction of +11.50/-1.00 x 75 right eye and +11.50/-1.00 x 65 left eye, her visual acuities were 6/12 right eye and 6/9 left eye, and subsequent secondary intraocular lens insertion was planned. This case highlights the importance of a careful review of the whole eye to ensure that remediable causes of visual loss are not missed.

## Introduction

Cataract refers to opacification of the lens of the eye and is considered by many to be a normal consequence of aging. Following development of visually significant symptoms, the standard management of a cataract is to remove the opacified lens by phacoemulsification and to insert a synthetic intraocular lens (IOL) within the capsular bag [1]. Estimates suggest that by the age of 75, over half of the given population is likely to have developed cataracts. It follows therefore that surgery to restore vision following development of cataracts is one of the most performed operations in healthcare [2]. Despite the development of a good surgical solution, cataract remains a leading cause of blindness, mostly owing to the demand of surgery and the limited access to the necessary resources worldwide [3]. The cost of surgery is decreasing, and the indication for phacoemulsification and IOL insertion is widening. For example, glaucoma surgeons are increasingly performing phacoemulsification with IOL insertion to increase the drainage of aqueous humor from the anterior chamber thereby lowering intraocular pressure [4]. Given these factors, rates of phacoemulsification with secondary IOL insertion being performed are likely to increase with time and as such it is important to understand the complications that may result from this procedure [5,6].

Cataract surgery is widely known to have a low complication rate and to provide excellent visual outcomes for patients with only 0.1% of patients experiencing worse vision following the procedure [7]. One of the rare complications of surgery is dislocation of the IOL. These are classified as dislocation with or without the capsular bag. Dislocation of the IOL without the bag is more common in the early post-operative period and relates to weakening of the posterior capsule secondary to stress of surgery. Dislocation of the IOL with the bag tends to be a later complication and is often termed "spontaneous dislocation." This can occur years after the procedure. Diagnosing these patients can be difficult as they are likely to present with sudden and severe visual loss without recent surgery and so the possibility of a surgical complication may not be borne in mind [8].

In this case report we highlight the importance of a careful examination of the whole eye, particularly in complex cases. By a thorough examination of the peripheral retina in this case, despite having already had a diagnosis that seemed to explain the visual loss, a dislocated IOL was identified thereby allowing restoration of vision. This case report was previously presented as a poster at the 38th Congress of the European Society of Cataract and Refractive Surgeons on October 3, 2020.

## Case presentation

The patient presented with a sudden loss of right vision; she described noticing a blurring and a black area in the central vision. She denied any head or jaw ache or appetite loss. Her past ocular history included referral by her optometrist for management of diabetic macular edema 15 years previously. At that time and over the following four years she underwent six focal laser treatments to her left eye and three to the right eye with good response and resolution of the edema. She then developed proliferative diabetic retinopathy (PDR) requiring six applications of pan-retinal photocoagulation (PRP) on right eye and seven on left eye. Despite this, she developed bilateral rubeosis iridis. At 65 years of age, she underwent bilateral vitrectomies and endolaser. Bilateral cataracts developed over the next 18 months and she underwent successful bilateral phacoemulsification with posterior chamber intraocular lens implant surgery at the age of 66 years, followed by yttrium aluminum garnett (YAG) capsulotomies. Visual acuities post-operatively were 6/9 bilaterally until this presentation with a sudden reduction in her right vision. Her past medical history includes treating hepatitis C, poorly controlled type 1 diabetes mellitus onset at the age of 32 years, and variably controlled hypertension as well as hypothyroidism and peripheral neuropathy with a right Charcot’s foot.

**Figure 1:**
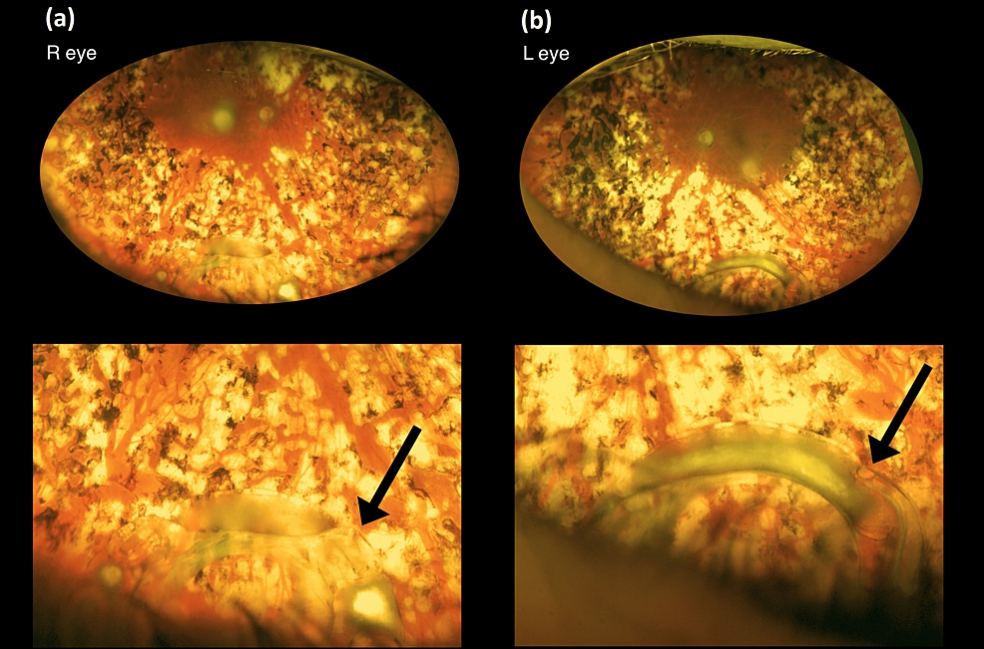
Both dislocated intraocular lenses (a and b) are seen encased in the capsular bag (arrows). Pale optic disc nerve and widespread pan-retinal photocoagulation can also be seen.

Examination at the time of presentation of reduced right vision revealed visual acuities of counting fingers (CF) right eye and 6/24 pinhole 6/12 left eye; both pupils were very small and unreactive, but no rubeosis. Anterior segments were unremarkable with a clear view, and intraocular pressure was 17 mmHg in both eyes. Retinal examination revealed pre-existing pan-retinal photocoagulation scars, pale discs, and no macular edema on fundoscopy. Blood pressure was 191/111 mmHg (known to be very variable and under review), there were no focal neurological signs, and the erythrocyte sedimentation rate (ESR) was normal. The most likely diagnosis was thought to be non-arteritic ischemic optic neuropathy. She was booked for carotid scans which revealed 50% stenosis bilaterally and a further appointment for reassessment was given. At that appointment, a full examination was performed again including a meticulous examination of the peripheral retina, which revealed a right dislocated intraocular lens on the inferior retina (Figures 1a, 1b). The left intraocular lens was in situ. Refraction improved the right vision from counting fingers to 6/36 pinhole 6/9. Shortly after this, she re-presented with a reduction in the left vision; examination revealed that the intraocular lens in the capsular bag was located on the inferior retina. Visual acuities were 6/12 right eye and 6/9 left eye with aphakic spectacles (+11.50/-1.00 x 75 right eye and +11.50/-1.00 x 65 left eye). She elected to go ahead with secondary lens implants; the dislocated intraocular lenses were left in situ. She achieved 6/18 right and left eyes. Fundoscopy and optical coherence tomography revealed epiretinal membranes in addition to the previously noted pan-retinal photocoagulation scars, pale discs, and attenuated vessels.

## Discussion

Spontaneous lens dislocation is a rare occurrence following cataract surgery and refers to dislocation of the IOL with its surrounding capsular bag. Following phacoemulsification, there is a 1.7% risk that lens dislocation will occur in the next 25 years [9]. Visual loss due to anterior lens dislocation is easier to diagnose, as the dislocated lens is visible. Pain is also likely to be present if acute secondary angle closure glaucoma occurs [10]. Spontaneous lens dislocation most commonly occurs six to 12 years after a cataract procedure with a mean patient age of 75 years, however, there is not thought to be a gender preponderance [11]. The main mechanisms thought to contribute to lens dislocation are zonular dehiscence, contraction of the capsular bag, and trauma. In fact, a study by Jakobsson et al. in 2010 showed that a third of patients with spontaneous IOL dislocation had some degree of zonular dehiscence at the time of surgery [12].

Pseudoexfoliation syndrome has been established as one of the most important predisposing factors and is present in approximately half of the cases of IOL dislocation [11]. It is characterized by the accumulation of microscopic granular amyloid-like proteins. An association between spontaneous lens dislocation and glaucoma has also been shown [13]. Diabetic eye disease is a risk factor for lens dislocation, and this is thought to be the case due to its association with contraction of the anterior capsule following phacoemulsification [8]. Prior vitreoretinal surgery, myopia, retinitis pigmentosa, and uveitis have also all been associated with increased risk of IOL dislocation [14-16].

Once the patient is diagnosed with spontaneous dislocation, management depends on the degree to which the symptoms are causing visual disturbance. In cases with partial subluxation of the IOL, the visual consequences may be negligible and so a conservative approach may be adopted. In cases of complete dislocation with the IOL in the vitreous cavity, the visual impairment is likely to be highly significant thereby justifying surgical intervention. Regarding optimal timings of surgery, Ostern et al. found that complications increased by 36% in patients whose repair operation was delayed by more than one month, suggesting prompt management to be in the best interests of the patient [17].

Our patient had bilateral phacoemulsification and YAG capsulotomy as well as bilateral vitrectomies on a background of long-standing diabetic eye disease. In our case, it is possible that the dislocation occurred as a result of the YAG laser capsulotomies, but these were performed nine years before the dislocations occurred and there was no history of ocular trauma. As such, spontaneous lens dislocation is a more likely diagnosis. The discovery of the dislocated lenses was made while examining the peripheral retina in careful detail. The intraocular lenses were quite peripheral and would not have been seen without dilation and specific examination of the far periphery, as she had small pupils. The discovery of a remediable cause of the catastrophic visual loss made an enormous difference to the patient who had started to put in place social support and lifestyle changes.

## Conclusions

In this patient with a history of visual obscuration, pale discs, significant vascular risk factors, uncontrolled blood pressure, and poorly controlled diabetes, the diagnosis of anterior ischemic optic neuropathy (AION) was made initially. By performing a meticulous peripheral retinal examination at the follow-up appointment the cause of the visual loss was identified. The reduction in her vision due to the dislocated lenses had a significant impact on her quality of life. This case highlights the importance of a careful comprehensive ocular examination and keeping all potential diagnoses in mind when examining patients with unexplained visual loss.
